# Network pharmacology: a crucial approach in traditional Chinese medicine research

**DOI:** 10.1186/s13020-024-01056-z

**Published:** 2025-01-12

**Authors:** Yiyan Zhai, Liu Liu, Fanqin Zhang, Xiaodong Chen, Haojia Wang, Jiying Zhou, Keyan Chai, Jiangying Liu, Huiling Lei, Peiying Lu, Meiling Guo, Jincheng Guo, Jiarui Wu

**Affiliations:** 1 School of Chinese Materia Medica, Beijing University of Chinese Medicine, Beijing, 100029 China; 2 School of Chinese Medicine, Beijing University of Chinese Medicine, Beijing, 100029 China

**Keywords:** Network pharmacology, Traditional Chinese medicine, Database, Current state of research, Precision network pharmacology

## Abstract

Network pharmacology plays a pivotal role in systems biology, bridging the gap between traditional Chinese medicine (TCM) theory and contemporary pharmacological research. Network pharmacology enables researchers to construct multilayered networks that systematically elucidate TCM’s multi-component, multi-target mechanisms of action. This review summarizes key databases commonly used in network pharmacology, including those focused on herbs, components, diseases, and dedicated platforms for network pharmacology analysis. Additionally, we explore the growing use of network pharmacology in TCM, citing literature from Web of Science, PubMed, and CNKI over the past two decades with keywords like “network pharmacology”, “TCM network pharmacology”, and “herb network pharmacology”. The application of network pharmacology in TCM is widespread, covering areas such as identifying the material basis of TCM efficacy, unraveling mechanisms of action, and evaluating toxicity, safety, and novel drug development. However, challenges remain, such as the lack of standardized data collection across databases and insufficient consideration of processed herbs in research. Questions also persist regarding the reliability of study outcomes. This review aims to offer valuable insights and reference points to guide future research in precision TCM network pharmacology.

## Introduction

In October 2007, British pharmacologist Andrew L. Hopkins first introduced the concept of “network pharmacology” in Nature Biotechnology, establishing it as a specialized branch of pharmacology that utilizes biological network approaches to analyze the synergistic interactions between drugs, diseases, and therapeutic targets, focusing on “multi-target, multi-pathway” mechanisms [1, 2]. According to Hopkins, drugs exert their therapeutic effects through interactions among multiple targets within biological networks. Network pharmacology has emerged as a natural extension of life sciences, advancing towards systems biology. It synthesizes elements from systems biology, bioinformatics, and computational network science to analyze the complex molecular relationships between drugs and the human body from a systemic perspective, offering insights into comprehensive pharmacological mechanisms. This integrative approach now plays a pivotal role in guiding both new drug development and clinical practices. As a cutting-edge discipline, it leverages artificial intelligence (AI) and big data to drive more systematic approaches in pharmaceutical research [3–5].

Network pharmacology emphasizes the importance of biological network equilibrium and perturbation, asserting that the fundamental cause of disease is network imbalance [6]. Key technologies in network pharmacology analysis include random network generation and comparison, network stratification and clustering, network visualization, and network topology analysis. Network topology analysis specifically involves parameters like degree, betweenness, shortest path, central nodes, and modularity. By leveraging network topology analysis, researchers can examine multi-layered networks to isolate critical chemical components and core targets, allowing for the prediction of essential drug components and therapeutic targets. This approach enables a thorough clarification of the underlying mechanisms of drug action [7, 8].

Traditional Chinese medicine (TCM) is a profound cultural heritage of China, known for its comprehensive and systematic approach to healthcare. What set TCM apart is its emphasis on treating the human body as an integrated system, a holistic principle that defines its diagnostic and therapeutic methods. According to TCM theory, the body functions as a complex, interconnected system, and diseases emerge from the imbalance caused by the interaction of multiple factors. Illness disrupts this intricate network, leading to an imbalance that underlies the development of disease. TCM uses herbal formulas based on the principles of “the seven relations of medicinal compatibility” and the roles of “chief, deputy, assistant, and envoy”. This approach customizes treatment according to individual patient presentations—whether cold, heat, warmth, or coolness—targeting specific symptoms to restore balance and optimize the body’s functional networks [9]. As TCM continues to gain global recognition, its integration with modern scientific methods remains a key challenge. The traditional research model, focused on “single target, single disease, and single drug” has long been the standard in pharmacology. However, this reductionist approach fails to address the multi-component, multi-target mechanisms of TCM formulas, which presents an ongoing challenge for researchers [10]. To address this gap, innovative approaches are needed to better understand the scientific mechanisms underlying TCM’s therapeutic effects, bridging the divide between traditional practices and modern scientific understanding.

**Fig. 1:**
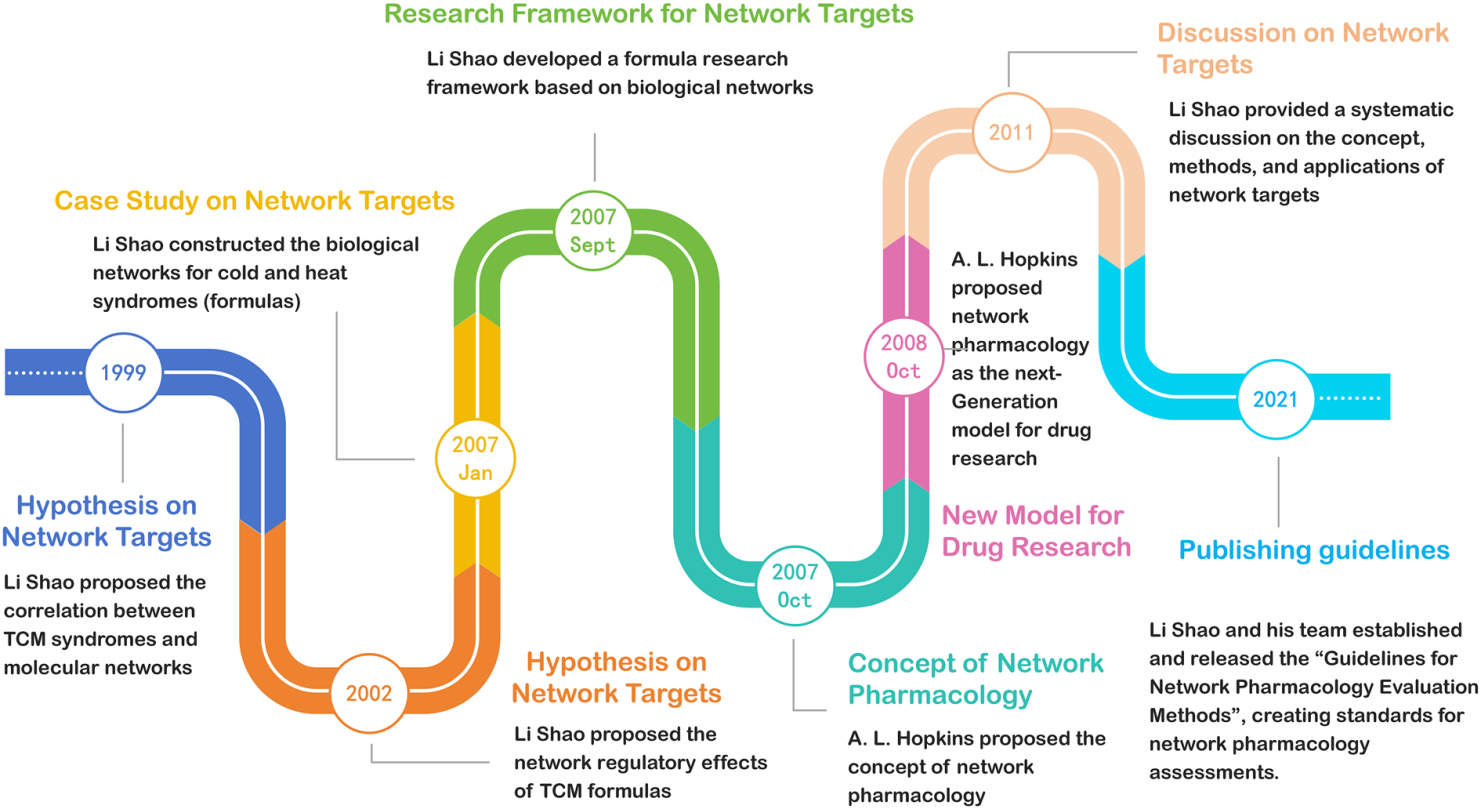
Timeline of network pharmacology development

The integration of TCM and network pharmacology dates back to the 1990s (Fig. 1). In 1999, Li Shao from Tsinghua University introduced the hypothesis linking TCM and biomolecular networks at the annual meeting of the China Association for Science and Technology, marking the beginning of research in TCM network pharmacology [11]. Since 2002, Li Shao’s team has pioneered research from a systematic network perspective, exploring the biomolecular networks underlying TCM syndromes and how these networks are modulated by the herbal formulas. They were the first to illustrate the biomolecular networks of cold and heat syndromes and the regulatory impact of corresponding herbal formulas. Their findings uncovered a modular relationship linking TCM phenotypes, molecular mechanisms, and herbal treatments. They clarified the pathway of herbs leading to active components, which then interact with biological targets to influence diseases, providing insight into the synergistic multi-component and multi-target regulatory mechanisms of herbal formulas in disease treatment [12–14]. Extensive practice over the years has demonstrated that network pharmacology shares core principles with TCM. The alignment between network pharmacology’s systems-based methodology and TCM’s foundational holistic theories has proven highly complementary [15, 16]. A comprehensive literature review spanning the past two decades was conducted using the keywords “network pharmacology”, “TCM network pharmacology”, and “herb network pharmacology” across major databases such as Web of Science, PubMed, and CNKI. The findings indicate that network pharmacology has been extensively applied to explore and elucidate the pharmacological mechanisms underlying traditional Chinese medicine (TCM) [17–19].

This review aims to provide a comprehensive overview of the databases and methodologies used in network pharmacology, as well as its applications in exploring the active components, mechanisms of action, safety, and development of new drugs from TCM, offering a reference for future research in the field.

## Common databases in TCM network pharmacology

Network pharmacology research relies heavily on a diverse array of databases. In recent years, the rapid advancement of big data and AI has led to the emergence of numerous databases. Most of these databases encompass a vast collection of foundational experimental data, along with potential chemical components and target information predicted through various algorithms [20]. These databases form the bedrock of network pharmacology studies and can be categorized into herbal databases (Table 1), chemical component databases (Table 2), disease databases (Table 3), and network pharmacology analysis platforms (Table 4). This section offers an overview of the key content and functions of frequently utilized databases in network pharmacology research.

### Herbal databases

Traditional Chinese Medicine Systems Pharmacology Database and Analysis Platform (TCMSP) [21] includes 500 varieties of herbs from 38 categories as outlined in the 2010 edition of the “Chinese Pharmacopoeia”. It provides related chemical components and pharmacokinetic characteristics such as oral bioavailability (OB), drug similarity (DL), permeability in intestinal epithelial cells, the blood–brain barrier, and water solubility, alongside 3,339 potential targets sourced from the DrugBank database. Accessible at https://tcmsp-e.com/, it supports data queries related to herbs, chemical components, targets, and diseases, enabling users to delve into detailed pages via the corresponding TCMSP ID. One notable feature of TCMSP is its capability to screen and analyze chemical components, allowing users to filter pharmacokinetic parameters like OB and DL to identify target chemical components and their corresponding targets. The filtered information can then be imported into Cytoscape software for visual network analysis.

The Encyclopedia of Traditional Chinese Medicine (ETCM) [22, 23] encompasses 403 kinds of herbs from the 2015 edition of the “Chinese Pharmacopoeia”, along with 3,962 Chinese herbal formulations approved by the China National Medical Product Administration, 7274 chemical components obtained through manual retrieval from the PubChem database, and 3,027 disease entries sourced from HPO, OMIM, DisGeNET, and ORPHANET databases. Available at http://www.tcmip.cn/ETCM/, it offers comprehensive information on herbs, Chinese herbal formulations, chemical components, and corresponding targets, along with GO and KEGG enrichment analysis functions. Users can also predict new drug target information based on known chemical structures, and ETCM includes a systematic analysis function to explore relationships between herbs, formulations, components, gene targets, relevant pathways, and diseases, thereby establishing connected entity networks.

Symptom Mapping (SymMap) [24] includes 499 kinds of herbs from the 2015 edition of the “Chinese Pharmacopoeia”, 1717 manually organized and standardized Chinese medicine symptoms, 961 Western medicine symptoms mapped to Chinese medicine symptoms via the UMLS database, 19,595 drug components from TCMID, TCMSP, and TCM-ID databases, 4302 drug targets from HIT, TCMSP, HPO, DrugBank, and NCBI databases, and 5235 diseases from OMIM, MeSH, and Orphant databases. SymMap contains 6638 connections between herbs and Chinese medicine symptoms, 2978 connections between Western medicine and TCM symptoms, 12,107 connections between Western medicine symptoms and diseases, and 48,372 connections between herbs and components. Available at http://www.symmap.org/, it serves as an integrative database focused on the interplay between TCM and Western medicine. Users can browse and download entity information and interaction relationships from the homepage. Furthermore, the SymMap database presents an association network between entities, where information can also be filtered by parameters such as P values, FDRs (BH), and FDR (Bonferroni).

A Bioinformatics Analysis Tool for Molecular Mechanism of Traditional Chinese Medicine (BATMAN-TCM) [25] features 54,832 Chinese herbal formulations from TCMID, TCM-Suite, LTM-TCM, and ITCM databases, 8404 herbs from TCMID, TCM-Suite, HERB, and ITCM databases, 39,171 chemical components from TCMID, TCM-Suite, HERB, and ITCM databases, 9927 target proteins from the SWISS-PROT database, 217 pathways from the KEGG database, 11,931 functional entries from the Gene Ontology database, 5128 diseases from the OMIM database, and 1504 disease entries from the TTD database. Accessible at http://bionet.ncpsb.org.cn/, it is dedicated to analyzing the action mechanisms of medicine, featuring functions such as chemical component target prediction, functional analysis, and visualization of component–target–disease/pathway networks. Users can input herbal formulations, herb names, PubChem IDs, or InChIKey codes, and BATMAN-TCM will automatically retrieve constituent compounds and targets for further analysis. Additionally, it supports multi-threaded analysis, enabling users to submit multiple tasks, with a Venn diagram displaying target comparisons between tasks and enrichment results shown in a consolidated manner on the functional enrichment analysis page.

Traditional Chinese Medicine Integrative Database (TCMID) [26] compiles 46,914 herbal formulations from literature mining, 8159 herbs from the TCM-ID database and literature sources, 25,210 chemical components of from TCM-ID, HIT, TCM@Taiwan databases, and literature mining, 17,521 targets from HIT, STITCH, OMIM, FrugBank, and literature, 3791 disease entries from the OMIM database, and 6,826 drugs from the DrugBank database. Accessible at http: //wwwmegabionet.org/tcmid/, it aims to establish connections between disease targets and those of herbs, inferring potential therapeutic targets. Its primary focus is on network visualization and predicting unknown drug targets. If two chemical components interact with the same protein or different proteins based on the network, users can infer potential synergistic or antagonistic effects. If a component of herb interacts with a disease target protein, it suggests therapeutic mechanisms, and if the components share action targets with drugs in the DrugBank database, it infers potential therapeutic targets.

A High-throughput Experiment-and Reference-guided Database of Traditional Chinese Medicine (HERB) [27] includes 7263 kinds of herbs and their processed products from SymMap, TCMID, TCMSP, and TCM-ID databases, 49,258 chemical components, 1037 high-throughput sequencing experimental data from the NCBI GEO database, and 1966 references related to herbs and its components from the past decade, along with 12,933 targets from SymMap, HIT, TCMSP, and TCMID databases, and 28,212 diseases from the DisGetNet database. Accessible at,http://herb.ac.cn/ it integrates high-throughput experimental data with literature data mining, offering functions like browsing, searching, viewing, and downloading data related to herbs, components, target genes, diseases, high-throughput experiments, and reference data, while displaying GO and KEGG enrichment results. The high-throughput data within HERB is also visualized, allowing users to select different datasets for display.

Similar databases include TCM Database@TaiWan [28], which features 443 herbs, over 20,000 compounds, and their three-dimensional structural information. Accessible at http://tcm.cmu.edu.tw/, it enables docking of small molecule drugs with macromolecules for computer-aided drug design. The Traditional Chinese Medicine Information Database (TCM-ID) [29], developed by the National University of Singapore, includes 1588 herbal formulations, 1313 herbs, 5669 chemical components, and 3725 three-dimensional structures. Available at https://www.bidd.group/TCMID/, it supports drug target enrichment analysis and visualization of gene expression from individual patient samples. Herbal Ingredients’ Targets Database (HIT) [30] features 1250 varieties of herbs, 1237 chemical components, 2208 targets, 10,031 compound-target activity pairs, 1231 therapeutic targets, and 56 micro-RNA targets. Accessible at http://www.badd-cao.net:2345/, it supports drug target prediction alongside querying functions.

Databases such as TCMSP, ETCM, and HERB represent some of the most widely recognized resources for herbal information. TCMSP exclusively catalogs plant-based herbs, omitting data on minerals and animal-derived substances. In contrast, HERB is considered one of the most comprehensive herbal repositories, incorporating a broad spectrum of data on plant-derived, animal-based, and mineral-origin herbs, alongside information on processed herbal products. Furthermore, specialized databases like SymMap, BATMAN-TCM, and TCMID extend beyond basic herbal data, emphasizing the intricate associations between herbs and various diseases.

**Table 1:** Herb databases related to network pharmacology

Database	URL	Description	Function	Refs
TCMSP (Traditional Chinese Medicine Systems Pharmacology Database and Analysis Platform)	https://tcmsp-e.com/	TCMSP comprises data on 500 herbs, their associated chemical compounds, ADME characteristics, and 3,339 potential targets	– Provides search and download functionalities for herbs, chemical compounds, target, and disease-related information	[21]
ETCM (The Encyclopedia of Traditional Chinese Medicine)	http://www.tcmip.cn/ETCM/	ETCM contains information on 403 herbs, 3,962 formulas, 7,274 chemical compounds, and 3,027 diseases	– Enables searches for herbs, formulas, chemical compounds, targets, and disease data – Predicts novel drug targets based on known drug structures – Constructs complex networks linking herbs, formulas, compounds, targets, pathways, and diseases	[22]
SymMap (Symptom Mapping)	http://www.symmap.org/	SymMap integrates 499 herbs, 1,717 manually recorded TCM symptoms, 961 Western medicine symptoms mapped to TCM symptoms, 19,595 chemical compounds, 4,302 targets, and 5,235 diseases	– Facilitates searches for relationships among herbs, chemical compounds, drug targets, TCM symptoms, Western medicine symptoms, and associated diseases – Visualizes complex networks among these entities	[24]
BATMAN-TCM (A Bioinformatics Analysis Tool for Molecular Mechanism of Traditional Chinese Medicine)	http://bionet.ncpsb.org.cn/	BATMAN-TCM includes data on 54,832 formulas, 8,404 herbs, 39,171 chemical compounds, 9,927 target proteins, 217 pathways, 11,931 GO terms, and 6,632 diseases	– Facilitates searches for formulas, herbs, and chemical compounds – Predicts targets of chemical compounds in herbs – Conducts functional enrichment analysis of targets – Visualizes networks of compounds–target–disease/pathway relationships	[25]
TCMID (Traditional Chinese Medicine Integrative Database)	http://www.megabionet.org/tcmid/	TCMID integrates 46,914 formulas, 8,159 herbs, 25,210 chemical compounds, 17,521 targets, 3,791 diseases, and 6,826 monomers	– Provides searches for formulas, herbs, chemical compounds, targets, and disease data – Visualizes herb–disease, compound–target, and compound–target–disease–herb networks – Predicts unknown drug targets	[26]
HERB (A High-throughput Experiment-and Reference-guided Database of Traditional Chinese Medicine)	http://herb.ac.cn/	HERB includes data on 7,263 herbs, 49,258 chemical compounds, 1,037 high-throughput sequencing datasets, 1,966 reference citations from the past decade, 12,933 targets, and 28,212 diseases	– Enables searches for herbs, active ingredients, target genes, diseases, high–throughput experiments, and references – Conducts herb–target enrichment analysis	[27]
TCM Database @ TaiWan	http://tcm.cmu.edu.tw/	TCM Database@TaiWan includes 443 herbs and over 20,000 chemical compounds with their 3D structure information	– Provides searches for herbs and chemical compounds – Offers 3D structural data of chemical compounds – Enables molecular docking between small molecules and macromolecular proteins	[28]
TCM-ID (Traditional Chinese Medicine Information Database)	https://www.bidd.group/TCMID/	TCM-ID includes 7,443 formulas, 2,751 herbs, 7,375 chemical compounds, 768 targets, 366 TCM syndromes, 2,756 GO terms, 222 KEGG pathways, and 27,716 disease sequencing samples	– Provides searches for formulas, TCMs, chemical compounds, targets, and disease data – Conducts drug–target enrichment analysis – Displays gene expression data from individual patient samples	[29]
HIT (Herbal Ingredients’ Targets Database)	http://www.badd-cao.net:2345/	HIT contains data on 1,250 herbs, 1,237 chemical compounds, 2,208 targets, 10,031 compound–target activity pairs, 1,231 targets, and 56 microRNA targets	– Facilitates searches for herbs, compounds, and targets – Predicts drug targets	[30]

### Chemical component databases

PubChem (https://pubchem.ncbi.nlm.nih.gov/) [31] is an extensive database that encompasses 118,596,691 compounds, 322,395,335 substances, and 295,360,133 biological activities, along with 41,558,769 literature references, 113,242 gene records, 248,298 protein entries, and 241,163 pathway details. It facilitates the retrieval of compounds through various identifiers such as names and molecular formulas and supports the download of both 2D and 3D structural representations. Users can find detailed information regarding the chemical and physical properties, biological activities, safety and toxicity, patents, and literature citations.

Swiss ADME (http://www.swissadme.ch/) [32] serves as a small molecule drug design platform, enabling researchers to compute physicochemical descriptors and predict ADME parameters, pharmacokinetic properties, drug-like characteristics, and the medicinal chemistry friendliness of single or multiple small molecules, thereby aiding in drug discovery efforts.

ChEMBL (https://www.ebi.ac.uk/) [33] features 2,431,025 distinct compounds, nearly 16,000 targets, and 20,772,701 recorded activities, along with 89,892 publications and 262 deposited datasets. This platform allows users to retrieve comprehensive information about compounds and targets, predict therapeutic targets, and search for structurally similar compounds based on specific structures.

The Drug-Gene Interaction Database (DGIdb) (https://www.dgidb.org/) [34] hosts data on over 10,000 genes and 20,000 drugs linked to nearly 70,000 drug-gene interactions, classified into 43 potentially druggable gene categories. Users can explore drug–gene interactions and input lists of genes to identify all known or potentially druggable genes within their specified sets.

The Comparative Toxicogenomics Database (CTD) (https://ctdbase.org/) [35] includes information on 17,100 chemicals, 54,300 genes, 6100 phenotypes, 7270 diseases, and 202,000 exposure statements. It allows users to investigate the chemical–gene/protein interactions, chemical–disease relationships, and gene-disease relationships.

Search Tool for Interactions of Chemicals Dataset (STTTCH) (http://stitch.embl.de/) [36] is designed for predicting interaction relationships between chemicals and genes. Users can input individual or multiple genes/chemicals, chemical structures, or protein sequences to ascertain predicted chemical compound–gene interactions.

DrugCentral (https://drugcentral.org/) [37] features 4927 active chemical compounds and 112,359 FDA-approved drugs, providing comprehensive information on active ingredients, chemical entities, drug products, mechanisms of action, indications, and pharmacological properties. It also supports drug similarity searches.

DrugBank (https://go.drugbank.com/) [38] hosts a comprehensive database containing 4563 FDA-approved drugs, 6231 investigational compounds, 6231 drug–drug interactions, 2475 drug–food interactions, and 5236 drug-related targets, alongside thousands of pathways. It serves as a powerful tool for searching information on drugs, targets, pathways, and indications, as well as for querying drug–drug and drug–food interactions.

PubChem is the world’s largest repository of chemical compounds, offering comprehensive access to virtually any desired compound. DrugBank, on the other hand, focuses predominantly on chemical drugs, with limited representation of natural small-molecule compounds. Swiss ADME stands out for its predictive capabilities, enabling researchers to estimate the ADME (Absorption, Distribution, Metabolism, and Excretion) properties of input chemical compounds. Additional databases, including ChEMBL, DGIdb, and CTD, are valuable resources for retrieving detailed information on chemical compounds and their molecular targets. Selecting an appropriate chemical database should align with the specific goals of the research being conducted.

**Table 2:** Chemical component databases related to network pharmacology

Database	URL	Description	Function	Refs
PubChem	https://pubchem.ncbi.nlm.nih.gov/	encompasses 118,596,691 compounds, 322,395,335 substances, and 295,360,133 biological activities, along with 41,558,769 literature references, 113,242 gene records, 248,298 protein entries, and 241,163 pathway details	– Search for compounds using their names, molecular formulas, or structures – Download 2D and 3D structures of compounds – Access chemical and physical properties, biological activities, safety and toxicity information, patents, literature citations, and more	[31]
Swiss ADME	http://www.swissadme.ch/	A small-molecule drug design platform	– This platform enables the computation of physicochemical descriptors and predictions of ADME parameters, pharmacokinetics, drug–likeness, and medicinal chemistry friendliness for one or more small molecules, supporting drug discovery processes	[32]
ChEMBL	https://www.ebi.ac.uk/	ChEMBL contains data on 2,431,025 distinct compounds, 15,598 targets, 20,772,701 bioactivities, 89,892 publications, and 262 deposited datasets	– Search for information on compounds and targets – Predict therapeutic targets – Identify structurally similar compounds based on a given molecular structure	[33]
DGIdb (Drug-Gene Interaction Database)	https://www.dgidb.org/	DGIdb catalogs over 10,000 genes and 20,000 drugs involved in nearly 70,000 drug-gene interactions or classified into 43 potentially druggable gene categories	– Explore drug–gene interactions – Users can input a list of genes to retrieve known or potentially druggable genes	[34]
CTD (Comparative Toxicogenomics Database)	https://ctdbase.org/	CTD includes data on 17,100 chemicals, 54,300 genes, 6,100 phenotypes, 7,270 diseases, and 202,000 exposure statements	– Provides searchable information on chemical–gene/protein interactions, chemical–disease relationships, and gene–disease associations	[35]
STITCH (Search Tool for Interactions of Chemicals Dataset)	http://stitch.embl.de/	A platform for predicting interactions between chemicals and genes	– Input single genes/chemicals – Input multiple genes/chemicals – Input chemical structures – Input protein sequences	[36]
DrugCentral	https://drugcentral.org/	Contains 4,927 active chemical ingredients and 112,359 FDA-approved drugs	– Search for information on active ingredients, chemical entities, drug products, mechanisms of action, indications, and pharmacological functions – Explore drug similarity	[37]
DrugBank	https://go.drugbank.com/	DrugBank houses data on 4,563 FDA-approved drugs, 6,231 investigational drugs, 6,231 drug–drug interactions, 2,475 drug–food interactions, and 5,236 drug-related targets	– Search for information on drugs, targets, pathways, and indications – Investigate drug–drug and drug–food interactions	[38]

### Disease databases

DisGeNet (https://disgenet.cn/) [39] encompasses 5912 diseases, 1915 LncRNAs, 16,065 protein-coding genes, and 2611 MicroRNAs, alongside 447,382 interaction relationships. It enables users to query interactions between diseases and LncRNAs, protein-coding genes, and MicroRNAs while supporting specific disease-gene information retrieval and data downloads.

GeneCards (https://www.genecards.org/) [40] is a comprehensive database featuring 466,227 genes, including 43,850 HGNC-approved genes, 21,612 protein-coding genes, and 291,831 RNA genes, comprising 130,757 lncRNAs, 111,811 piRNAs, and 49,263 other ncRNAs. The database also lists 20,956 disease-associated genes and 128,261 functional elements. GeneCards facilitates the retrieval of disease-related targets, gene-specific information, and provides interactive pathway network maps.

The Online Mendelian Inheritance in Man (OMIM) database [41] features 17,375 gene descriptions, 14 combined gene and phenotype entries, 6895 phenotypes with known molecular bases, 1499 phenotypes with unknown molecular bases, and 1736 entries primarily relating to suspected Mendelian phenotypes. The OMIM database (https://www.omim.org/) allows users to query disease-related targets and gene-specific information.

Human Protein Reference Database (HPRD) [42] offers 30,047 protein entries, 41,327 protein–protein interactions, 93,710 post-translational modifications (PTMs), 112,158 protein expression datasets, 22,490 subcellular localizations, 470 domains, and 453,521 PubMed links. HPRD (http://www.hprd.org/) provides a wealth of protein annotation information, including expression profiles, classifications, and structural domains.

Databases like DisGeNet, GeneCards, and OMIM serve as rich repositories of disease-related target data, though their scope of coverage and data curation standards differ. To ensure a more comprehensive identification of disease-associated targets, researchers typically integrate data from multiple sources, leveraging the unique strengths of DisGeNet, GeneCards, and OMIM.

**Table 3:** Diseases databases related to network pharmacology

Database	URL	Description	Function	Refs
DisGeNET	https://disgenet.cn/	DisGeNET encompasses data on 5,912 diseases, 1,915 long non-coding RNAs (LncRNAs), 16,065 protein-coding genes, 2,611 microRNAs, and 447,382 interactions	– Explore interactions between diseases and LncRNAs, protein-coding genes, and microRNAs – Retrieve specific diseases and gene information – Download relevant datasets	[39]
GeneCards	https://www.genecards.org/	GeneCards hosts 466,227 genes, including 43,850 HGNC-approved genes, 21,612 protein-coding genes, and 291,831 RNA genes (comprising 130,757 lncRNAs, 111,811 piRNAs, and 49,263 other ncRNAs), as well as 20,956 disease-associated genes and 128,261 functional elements	– Identify disease-related targets – Access comprehensive gene information – View interactive pathway network maps	[40]
OMIM (Online Mendelian Inheritance in Man)	https://www.omim.org/	OMIM includes 17,375 gene descriptions, 14 gene–phenotype combinations, 6,895 phenotype descriptions with known molecular bases, 1,499 phenotype descriptions or loci with unknown molecular bases, and 1,736 additional phenotypes, mostly suspected of having a Mendelian inheritance basis	– Search for disease-related targets – Access detailed gene-related information	[41]
HPRD (Human Protein Reference Database)	http://www.hprd.org/	HPRD provides data on 30,047 protein entries, 41,327 protein–protein interactions, 93,710 post-translational modifications (PTMs), 112,158 protein expression profiles, 22,490 subcellular localizations, 470 domains, and 453,521 PubMed links	– Offers extensive protein annotations, including expression profiles, classifications, and structural domains	[42]

### Network pharmacology analysis platforms

STRING (https://cn.string-db.org/) [43] is a comprehensive database comprising 12,535 organisms and over 59.3 million proteins, encompassing approximately 20 billion documented protein interaction relationships. The platform enables users to retrieve information on individual or multiple proteins, construct protein–protein interaction networks, and conduct Gene Ontology (GO) and KEGG pathway enrichment analyses.

RCSB PDB (https://www.rcsb.org/) [44] hosts 224,931 protein structures and 1,068,577 computationally derived structural models, providing valuable access to the three-dimensional structures of various proteins, which is essential for understanding their functional mechanisms.

DAVID (https://david.ncifcrf.gov/) [45] and Metascape (https://metascape.org/) [46] serve as powerful platforms for GO and KEGG enrichment analyses, offering functionalities for Gene ID conversion alongside comprehensive enrichment analysis capabilities.

The Molecular Interaction Database (MINT) (https://mint.bio.uniroma2.it/) [47] consists of data on 607 species, encompassing 139,547 interaction relationships and 27,756 unique interactors, alongside 6425 related publications. MINT is designed to facilitate the exploration of protein–protein interaction relationships.

The Kyoto Encyclopedia of Genes and Genomes (KEGG) (https://www.kegg.jp/) [48] comprises 573 pathway maps, 201 functional hierarchies, 489 KEGG modules, and 48 reaction modules. This platform allows for extensive pathway retrieval and visualization, enabling users to search for specific pathways that involve particular genes by their gene names. In network pharmacology research, the STRING and MINT databases are widely utilized for constructing PPI networks. For GO functional enrichment analysis and KEGG pathway enrichment analysis, platforms such as DAVID and Metascape are frequently employed. The RCSB PDB database is indispensable for accessing three-dimensional protein structures, which are crucial for molecular docking studies. Given the diverse functionalities of network pharmacology analysis platforms, researchers are advised to choose the platform that best aligns with their specific research objectives.

**Table 4:** Network pharmacology analysis platforms and databases

Database	URL	Description	Function	Ref
STRING	https://cn.string-db.org/	STRING integrates data from 12,535 organisms, 59.3 million proteins, and over 20 billion protein–protein interactions	– Search for single or multiple proteins – Construct protein–protein interaction networks – Perform GO/KEGG enrichment analysis	[43]
RCSB PDB	https://www.rcsb.org/	Contains 224,931 protein structures and 1,068,577 computed structure models	– Provides access to detailed 3D protein structures	[44]
DAVID	https://david.ncifcrf.gov/	A platform for GO/KEGG enrichment analysis	– Functional annotation tools – Gene functional classification tools – Gene ID conversion tools – Gene name batch viewer	[45]
Metascape	https://metascape.org/	A GO/KEGG enrichment analysis platform	– Gene ID conversion – Enrichment analysis tools	[46]
MINT (Molecular Interaction Database)	https://mint.bio.uniroma2.it/	Includes data on 607 species, 139,547 interactions, 27,756 interactors, and 6,425 publications	– Provides insights into protein–protein interaction networks	[47]
KEGG (Kyoto Encyclopedia of Genes and Genomes)	https://www.kegg.jp/	Includes 573 pathway maps, 201 functional hierarchies, 489 KEGG modules, and 48 reaction modules	– Pathway search and visualization – Search for pathways by gene names	[48]

### Conclusion

The herbal, chemical compound, and disease databases, along with network pharmacology analysis platforms mentioned above, are integral tools in contemporary TCM network pharmacology research. Each database type offers unique data, as detailed in this discussion of their specific contents and functionalities. To enhance the reliability and robustness of network pharmacology outcomes, researchers are encouraged to synthesize data from multiple databases in conjunction with insights from existing literature.

## Current status of TCM network pharmacology research

**Fig. 2:**
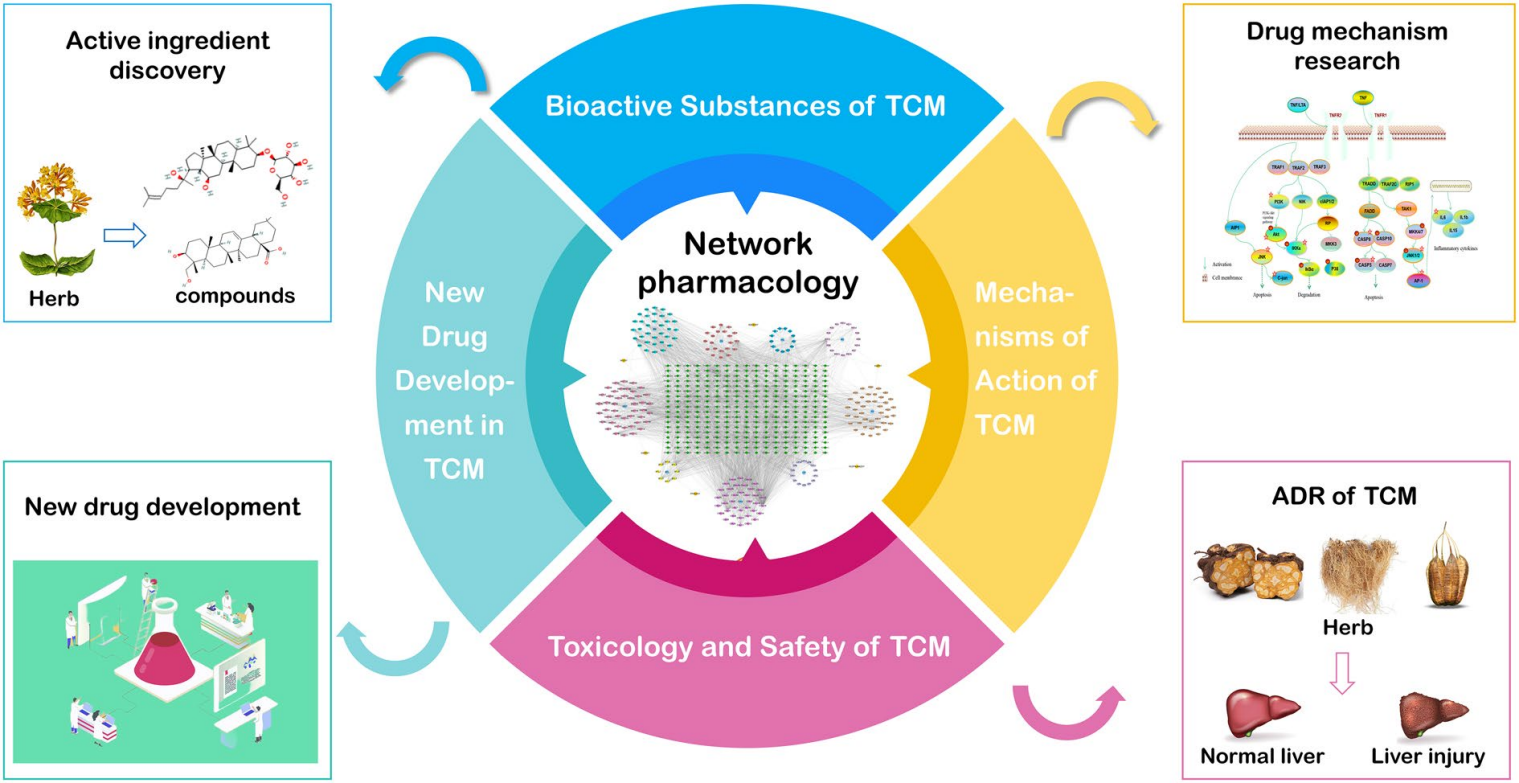
Schematic diagram of the application of network pharmacology in traditional Chinese medicine research

As research in TCM network pharmacology continues to advance, it becomes evident that the primary focus lies in four critical areas: the identification of the material basis of TCM efficacy, the elucidation of its mechanisms of action, the evaluation of TCM toxicology and safety, and the development of novel TCM drugs. This section will thoroughly explore these focal points, offering an in-depth analysis of the latest advancements in TCM network pharmacology (Fig. 2).

### Exploration of the material basis for the efficacy of TCM

The efficacy of TCM relies on a complex array of chemical substances within individual herbs or herbal formulas. Identifying specific effective components is challenging due to this complexity. Traditionally, research on the material basis of TCM efficacy follows a systematic process: extraction, separation, purification, structural identification, and experimental validation. While this methodology yields relatively reliable results, it is often time-consuming and less efficient [49].

In contrast, network pharmacology offers a novel approach by constructing networks that link drugs, compounds, targets, and diseases. This method allows for the mapping of potential chemical compounds to targets, elucidating the material basis for therapeutic effects and predicting key intervention targets. As network pharmacology becomes more prominent, it is increasingly employed to predict the efficacy material basis of TCM [50, 51]. For instance, Hou et al. [52] utilized network pharmacology to identify the effective components of Scutellaria Baicalensis in treating oral leukoplakia. Their work involved constructing protein–protein interaction (PPI) networks alongside “compound–target–disease” and “compound–target–pathway” networks, leading to the identification of 25 active chemical components and 31 key targets. Molecular docking further validated baicalein as a principal active ingredient. Similarly, Lou et al. [53] investigated the active constituents and mechanisms of Morodan, a TCM formulation for atrophic gastritis, through network pharmacology. They compiled a “drug–component–target” network that identified 724 potential active compounds and 961 targets, from which 179 potential therapeutic targets for atrophic gastritis were identified. Based on their degree of connectivity, the top 10 targets in the constructed PPI network were selected as core targets, which were then subjected to both in vivo and in vitro validation. Thakur et al. [54] researched *Thymus linearis* Benth. (TL) for gastrointestinal and neurological conditions, revealing that the 77 chemical components and 717 targets identified via network pharmacology were significantly linked to the neural ligand–receptor interaction pathways. Core therapeutic components identified included beta-citronellol, piperitol, p-cymen-8-ol, and alpha-humulene. The integration of high-performance liquid chromatography-mass spectrometry (HPLC–MS) with network pharmacology has significantly improved the accuracy of predicting active chemical components. Cao et al. [55] employed UPLC combined with network pharmacology to analyze the chemical components of citrus herbs, which primarily consist of flavonoids, phenolic acids, lignans, coumarins, and alkaloids. Notably, Chenpi (Citri Reticulatae Pericarpium) exhibit high levels of flavonoids, while Huajuhong (Citri Grandis Exocarpium) displays a rich presence of coumarins. Guang Chenpi shows significantly elevated levels of polymethoxyflavones. By utilizing HPLC–MS to detect components in blood or tissues and constructing a “component–target–network” diagram based on blood components, the actual pharmacokinetics of the drug in animal models can be accurately represented. Yan et al. [56] utilized UHPLC-Q-TOF–MS technology combined with network pharmacology to identify the effective components of Suanzaoren decoction for treating anxiety, identifying a total of 110 chemical constituents, including 20 that were found in blood circulation. They uncovered 21 serum biomarkers related to the metabolism of arachidonic acid, tryptophan, sphingolipid, and linoleic acid, constructing a comprehensive “chemical component–target–pathway” network that includes 11 effective components, 4 therapeutic targets, and 2 action pathways. Chen et al. [57] investigated the material basis and mechanisms of Xijiao Dihuang Tang (XDT) in addressing diseases associated with blood-heat and blood-stasis syndrome. Utilizing HPLC-QTQF-MS/MS, they identified 60 serum pharmacochemical constituents of XDT and predicted that these corresponded to 338 targets mainly involved in key biological processes such as inflammation, coagulation, cell proliferation, and apoptosis through network pharmacology. Bai et al. [58] examined the active components of Wuwei Shexiang pills for anti-gout effects. Their analysis using UPLC-Q/TOF–MS identified 38 active compounds, and network pharmacology in combination with molecular docking revealed 104 critical targets, with enrichment analysis indicating involvement in signaling pathways such as MAPK and NF-κB. Furthermore, deducing chemical components from targets obtained through omics sequencing has emerged as a viable strategy in network pharmacology. Zhu et al. [59] integrated network pharmacology with metabolomics and 16S rRNA sequencing to explore the effective components of Maca Compounds Prescription (MCP) against fatigue, yielding 120 active components and 116 fatigue-related targets. Gut microbiome analysis suggested that MCP could effectively reshape the intestinal microbial ecosystem. Zhao et al. [60] utilized network pharmacology to investigate the core chemical components and therapeutic targets of Kanglaite injection in treating triple-negative breast cancer, initially identifying 14 active compounds and 53 therapeutic targets from public databases. Through bioinformatics analyses, they further narrowed it down to 2 active compounds and 3 core targets. Subsequent in vitro experiments validated that Kanglaite injection could inhibit triple-negative breast cancer by blocking the cell cycle and downregulating CDK1 expression. Lastly, Shi et al. [61] reported on the components of Eggplant used for treating chilblains, identifying 2 natural compounds alongside 42 known compounds, of which 11 exhibited anti-inflammatory activity and 27 demonstrated antioxidant properties, primarily consisting of fatty acids, flavonoids, alkaloids, phenolic acids, saponins, and lignans.

### Mechanisms of action of TCM

TCM and its formula have received extensive global recognition for their clinical efficacy. The multi-component nature of TCM enables it to act on multiple pathways and targets simultaneously, enhancing its pharmacological efficacy in the treatment of various diseases. However, the understanding of TCM’s therapeutic advantages primarily relies on the accumulation of clinical experiences from previous practitioners. Modern scientific approaches often employ single-molecule biological techniques, which can only validate the mechanisms of TCM formulas at individual molecular levels, failing to elucidate the broader multi-pathway and multi-target mechanisms [62].

Network pharmacology offers a promising alternative by constructing a comprehensive biological network of “herbs–components–targets–pathways–diseases”. This framework allows for the analysis of various signaling pathways and target networks regulated by TCM, providing a systematic understanding of its overall mechanisms in disease management [63]. For the effective components and key targets identified through network pharmacology, molecular docking techniques can simulate the interactions between drug molecules and their targets at a computational level, facilitating result validation. Additionally, these findings can be confirmed through in vivo and in vitro experiments. Shang et al. [64] utilized network pharmacology to investigate the mechanisms underlying the action of Sijunzi Decoction in treating colorectal cancer. Their “component–target–disease” network analysis revealed 144 effective components, and 897 targets associated with Sijunzi Decoction. Molecular docking studies confirmed the binding affinities of core compounds with essential targets, while both cellular and animal model experiments demonstrated that Sijunzi Decoction primarily promotes apoptosis and autophagy in colorectal cancer cells by modulating the PI3K/Akt/mTOR signaling pathway, thereby achieving its therapeutic effects.

Zhou et al. [65] explored the action mechanisms of Morodan in the treatment of chronic atrophic gastritis using network pharmacology. They constructed a “herbs–biological functions” network, revealing that Morodan affects several biological functions, including cell proliferation and apoptosis, in the treatment of chronic atrophic gastritis. Subsequent cellular experiments validated that Morodan effectively inhibits the proliferation, apoptosis, and differentiation of GES-1 cells induced by 1-methyl-3-nitro-1-nitrosoguanidine while also reducing their inflammatory response. Chen et al. [66] investigated the mechanisms of berberine in treating hyperlipidemia. Their findings suggested that berberine regulates 13 metabolites, such as CE (16:1), HexCer (D18:1/19:0), and LPC (O-22:0), by targeting key pathways and mechanisms, including CHPT1, PLA2G4A, LCAT, and UGCG, through glycerophospholipid and sphingolipid pathways, thereby effectively treating hyperlipidemia. Jiao et al. [67] investigated the mechanisms by which the active component 6-shogaol in ginger exerts therapeutic effects on obesity. They found that 6-shogaol improves obesity by downregulating the expression of PPAR-γ and C/EBP-α and reducing the phosphorylation levels of IRS-1, PI3K, and AKT. Zhang et al. [68] studied the mechanisms underlying the efficacy of the proprietary Chinese medicine formula, Zhuanggu Busui Formula (ZGBSF), in treating osteoporosis. Their component–target–disease network analysis revealed that ZGBSF can act on 22 core targets to treat osteoporosis. Subsequent animal experiments confirmed that ZGBSF improves osteoporosis by upregulating the expression levels of Caspase-3, Bax, and Prap while downregulating the phosphorylation levels of Bcl-2, AKT1, and PI3K. Zhu et al. [69] explored the mechanisms of action of Xuanbai Chengqi Decoction (XCD) in the intervention of acute lung injury. Their component–target network identified 46 active components and 280 target genes of XCD. They further screened 753 differentially expressed genes (DEGs) from the acute lung injury model rats before and after administration using transcriptome sequencing. PPI network analysis indicated that VEGF, mTOR, AKT1, HIF-1α, and PI3K are core targets involved in the mechanism of action. Animal experiments demonstrated that XCD could downregulate the expression levels of TNF-α, IL-6, and IL-1β, alleviating pathological damage to lung tissue in rats, while also downregulating the protein expression levels of VEGF, mTOR, AKT1, HIF-1α, and PI3K to treat acute lung injury. Wang et al. [70] reported that Buyang Huanwu Decoction inhibits the progression of myocardial fibrosis by affecting the IL-17 signaling pathway, improving fibroblast function in rats, and reducing the expression of inflammatory factors. Xiong et al. [71] discovered that Zuojin Capsule improves precancerous gastric lesions by inhibiting the expression levels of cyclins CDK1, CCNB1, and CCNA2 in mice, suppressing the abnormal activation of the PI3K-AKT pathway and inducing cell cycle arrest at the G2/M phase. Li et al. [72] investigated the mechanisms of action of Citri Reticulatae Pericarpium (CRP) in treating type 2 diabetic osteoporosis. They constructed a “compound–target–disease” network revealing 5 chemical components and 63 therapeutic targets, with the PPI network analysis identifying AKT1, TP53, JUN, BCL2, MAPK1, NFKB1, and ESR1 as core targets of CRP. Enrichment analysis suggested that CRP primarily exerts therapeutic effects by regulating oxidative stress and hormone levels. Kong et al. [73] analyzed the mechanisms through which Hydroxysafflor Yellow A ameliorates liver aging in mice. They identified 199 potential targets through network pharmacology and 480 DEGs via transcriptome sequencing. By integrating the two approaches, they identified key targets including HSP90AA1, ATP2A1, NOS1, and CRAT, as well as critical pathways such as calcium signaling, estrogen signaling, and cGMP-PKG signaling. Finally, molecular docking was employed to validate the findings.

### Toxicology and safety research of TCM

TCM is generally considered to have fewer adverse reactions compared to chemical drugs when treating conditions such as tumors and diabetes [74, 75]. Reports on the adverse reactions associated with TCM are limited. In recent years, the combination of Chinese and Western medicine for treating complex diseases has become increasingly mainstream. However, this combination often leads to adverse reactions such as liver and kidney damage, which significantly compromise the effectiveness of clinical treatments. Conventional toxicological assessments of TCM rely on patient mortality rates, biochemical markers, and histopathological evaluations to determine safe and effective clinical dosages. Unfortunately, these assessments may not comprehensively address the toxicological bases of TCM, potentially overlooking harmful components present in certain formulas. Therefore, there is an urgent need to identify new methods for discovering toxic substances and assessing the safety of TCM [76–78].

Network pharmacology offers innovative approaches for investigating network toxicology and evaluating the safety of TCM. Network toxicology employs an interaction network involving “toxic components–targets–herbs” to analyze the toxicological bases of herbs, predict adverse drug reactions. Enrichment analyses generate “toxic component–target–pathway” networks to elucidate the mechanisms underlying adverse reactions [79]. Zhang et al. [80] reported that several herbs induce oxidative stress, by modulating key biomarkers such as SOD, MDA, GSH, ROS, GPx, Bax, caspase-3, Bcl-2, Nrf2, and NO, thereby causing liver damage. Jiang et al. [81] explored the chemical components and mechanisms of liver toxicity induced by Heshouwu (Polygoni Multiflori Radix) through a combination of network toxicology and spatial metabolomics. They constructed an interaction network linking 8 toxic components with their respective targets, and conducted molecular docking analyses based on enrichment results to evaluate the binding affinities of these toxic components with core targets. Their assessments, which included histopathological and serum biochemical evaluations in mouse models, indicated that the mechanisms behind Heshouwu’s hepatotoxicity are likely associated with lipid metabolism disorders, ROS, and mitochondrial damage. Cao et al. [82] applied network pharmacology to investigate the mechanisms underlying the hepatotoxicity induced by Shanglu (Phytolaccae Radix) in zebrafish larvae. The study revealed that the administration of Shanglu led to a significant increase in endogenous arachidonic acid levels, triggering oxidative stress responses. PCR analysis demonstrated a marked upregulation of Caspase-3, Caspase-8, and Caspase-9 expression, while Western blot results confirmed elevated Caspase-3 protein levels. These findings indicate that Shanglu disrupts amino acid metabolism in zebrafish, activates apoptosis-related proteins, and promotes hepatocyte apoptosis, shedding light on its hepatotoxic effects. He et al. [83] employed network pharmacology to elucidate the hepatotoxicity mechanism of Esculentoside A, a component of Shanglu. They identified 58 critical targets, including albumin, MAPK1, and Caspase-3, then analyzed 16 biomarkers, such as 5-hydroxykynurenamine and palmitic acid, in the blood of rats using metabolomics. Their findings suggest that Esculentoside A may induce liver damage by triggering oxidative stress and impacting energy metabolism, which in turn provokes inflammatory responses. Mesaconitine, found in Aconitum, is both an active compound and is associated with severe cardiotoxic and hepatotoxic effects. Chen et al. [84] investigated the mechanism of liver toxicity linked to Mesaconitine. Their network pharmacology analysis indicated that Mesaconitine modulates signaling pathways, including MAPK and HIF-1, through the regulation of key targets such as ALB, AKT1, CASP3, and IL2. Validation through molecular biology experiments confirmed that Mesaconitine may induce liver toxicity by activating oxidative stress pathways and triggering apoptosis.

### Research on new drug development in TCM

As the landscape of new drug development evolves, the traditional approach of designing highly selective drugs aimed at a single disease through a specific biological target may no longer be the most effective strategy [85–87]. Recent findings indicate that multiple drugs can often treat a single disease, and conversely, one drug may be effective against various diseases [88]. Network pharmacology, which integrates systems biology and multi-target pharmacology, transcends the limitations of single-target methodologies. It adopts a perspective in which a single chemical compound can interact with multiple targets, and different compounds may target the same biological pathway. This approach provides innovative strategies for the development of natural products and the repurposing of existing drugs [89]. By leveraging chemical component databases and employing machine learning or deep learning algorithms, researchers can predict the therapeutic potential of herbs with similar properties. This enhances the efficiency of drug discovery, reduces unnecessary experimental efforts, and broadens the selection of candidate herbs. For instance, Zhang et al. [90] identified common pathogenic targets for Alzheimer’s disease and rosacea using network pharmacology. Their enrichment analysis revealed that both conditions share pathways related to inflammation, metabolism, and apoptosis. They constructed a regulatory network of transcription factors, identifying 37 core transcription factors and target genes, and predicted 113 potential therapeutic agents using the DGIdb/CMap database. Notably, melatonin showed significant interactions with 19 of these key targets, and subsequent experiments confirmed its efficacy in regulating vascularization to treat both diseases.

The complex composition of proprietary Chinese medicines often complicates the assessment of their overall quality using singular indicators, posing challenges for regulatory approval. However, by employing network pharmacology to create a biological network connecting the medicines, their individual components, and associated diseases, researchers can more effectively identify key constituents that act as reliable indicators of clinical efficacy. This precision approach aids in the development of new proprietary Chinese medicine products. For example, Zhang et al. [91] combined experimental techniques with network pharmacology to develop a strategy for quality control in the “Danshen (Salvia miltiorrhiza) & Chuanxiong (Ligusticum chuanxiong)” herbal pair. They identified 9 critical chemical components, including tanshinone I, tanshinone IIA, cryptotanshinone, salvianolic acid B, ferulic acid, salvianolic acid A, rosmarinic acid, chlorogenic acid, and coniferyl ferulate, and confirmed their presence through HPLC analysis. Additionally, Ma et al. [92] employed chemometrics in conjunction with network pharmacology to investigate the pharmacological basis of Lanqin Oral Solution (LOS) in the treatment of pharyngitis. By correlating LOS efficacy with common fingerprint peaks, and using CARS and other chemometric techniques, they identified chromatographic peaks linked to therapeutic effects. Mass spectrometry analysis revealed the active components such as geniposide, berberine, palmatine, and baicalin. Network pharmacology was then used to construct a compound-target network, providing insights into the anti-inflammatory mechanisms of LOS and offering a novel strategy for its quality control. Furthermore, network pharmacology can integrate traditional medicinal knowledge with modern clinical research, facilitating network target prediction and experimental validation of key therapeutic processes in TCM formulas. Based on these findings, the herbal formulation can be optimized to address core symptoms [93].

## Discussion

### Challenges and solutions in network pharmacology research for TCM

Network pharmacology serves as a crucial methodology for elucidating the mechanisms underlying Chinese medicine. While network pharmacology has advanced fundamental research in TCM and yielded notable achievements, several limitations persist. The “herbs–components–targets–diseases” multi-level biological network established through network pharmacology effectively addresses the shortcomings of previous single-target studies. However, in many network pharmacology studies, the screening of active compounds is primarily guided by pharmacokinetic parameters such as OB and DL. This approach frequently disregards the actual abundance of these compounds in the herbal formula. As a result, the screened compounds may not represent the predominant or most therapeutically relevant constituents, and significant overlap in identified chemical components often occurs across different TCM formula. To enhance the reliability and clinical relevance of network pharmacology findings, researchers should integrate data from existing literature or perform liquid chromatography-mass spectrometry (LC–MS) analyses to quantify the concentrations of various chemical components within the investigational product of interest. This allows for the identification of key active compounds based on their relative abundance, thereby producing more robust and persuasive results. Moreover, Chinese medicine is predominantly administered after specific processing, which can significantly alter its chemical composition [94, 95]. The formula adheres to the TCM principles of monarch, minister, assistant, and envoy. The dosages of these herbs, particularly the monarch and minister, vary across prescriptions, and the combinations of different herbs can significantly influence the concentration of active components and the overall therapeutic efficacy. However, variations in chemical constituents pre- and post-processing, along with the differences in efficacy among various “herb pairs” in different formulas, are often not adequately captured in most databases, complicating the accurate representation of the true therapeutic effects of the formulas. Some researchers employing network pharmacology for target prediction tend to apply a mechanistic approach, simply overlaying the targets of various herbs within a formula. Nonetheless, factors such as the origin of the herbs, processing methods, and decoction techniques can all affect the effective components, thereby influencing the drug targets. Therefore, it is imperative to have a comprehensive understanding of the medicine being studied, integrating data from various databases and addressing existing gaps to improve the accuracy of predicting chemical components and their corresponding targets. Current disease databases generally overlook environmental factors and ethnic variations when predicting targets, which may result in suboptimal predictive accuracy. Furthermore, most databases incorporate disease nomenclatures aligned with modern medical perspectives, which may not always correspond with TCM syndromes. Given the rich practical insights offered by TCM, investigations into its mechanisms for treating specific diseases should explore the underlying connections between modern medical terminology and TCM syndromes. This approach could lead to the development of disease network models that align with TCM syndromes, thereby fostering an integration of network pharmacology with TCM theories. Ultimately, this holistic approach can provide a comprehensive understanding of the molecular mechanisms through which TCM exerts its therapeutic effects at the biological network level.

The absence of an evaluation framework for network pharmacology is a critical factor undermining the reliability of research in this field. In 2021, Li Shao and his team established and released the “Guidelines for Network Pharmacology Evaluation Methods”, creating standards for network pharmacology assessments. This initiative has standardized data usability in network pharmacology research, enhancing the reliability of findings [96].

### Integration of network pharmacology in TCM with AI and multi-omics: current trends

AI technology has significantly bolstered network pharmacology’s capacity to elucidate the molecular mechanisms by which TCM treats diseases. AI has been instrumental in addressing challenges in drug target discovery, investigating mechanisms of drug action, and identifying new applications for natural products [97, 98]. For instance, Keiser et al. [99] developed a drug target analysis methodology based on similarity computations, which compares the chemical structures of drugs with ligands that are known to modulate protein receptor functions. This approach facilitates the establishment of indirect links between drugs and their targets through these ligands. Additionally, Li et al. [100] introduced a target prediction algorithm called drugCIPHER, which utilizes a holistic association of “drug network–molecular network”. This algorithm predicts drug–target interactions across the genome by analyzing modular relationships observed in pharmacology and genomics. With the ongoing accumulation of multidimensional omics data—including drug molecular structures, therapeutic applications, adverse effects, and laboratory test results—a robust big data foundation is being established in the field of network pharmacology, thereby creating new avenues for AI technologies.

Having evolved over the past decade, network pharmacology is increasingly integrating with multi-omics technologies, such as transcriptomics, proteomics, and metabolomics. This integration is being widely applied in fundamental research areas, including the pharmacological mechanisms of Chinese medicinal formulas, the development of new Chinese medicines, and the investigation of the material basis of TCM efficacy [101]. For example, research by Ye et al. [102] utilized metabolomics and transcriptomics in conjunction with network pharmacology to reveal that the Dengzhan Shenmai capsule can regulate glutamate levels by influencing the citric acid cycle and glutamate-related pathways. This regulation results in the inhibition of the NF-κB signaling pathway, thus providing anti-inflammatory effects and neuroprotection in the treatment of ischemic stroke. In another study, Liu et al. [103] combined transcriptomics and proteomics to analyze peripheral blood mononuclear cells from HIV immunological non-responders (INR) treated with the Tripterygium Wilfordii Hook F (TwHF) pill. This analysis identified the core targets and pathways modulated by TwHF, leading to the construction of a “TwHF–compounds–targets–INR” network through network pharmacology. Their predictions identified STAT1 and triptolide as central targets, and subsequent cellular experiments demonstrated that the primary component of TwHF, triptolide, exerts therapeutic effects by inhibiting the activation of immune cells, impacting the IFN signaling pathway, and consequently reducing the production of IFNγ, the expression of downstream IFN-stimulated genes, and the phosphorylation of STAT1.

### Precision network pharmacology: a new research paradigm

**Fig. 3:**
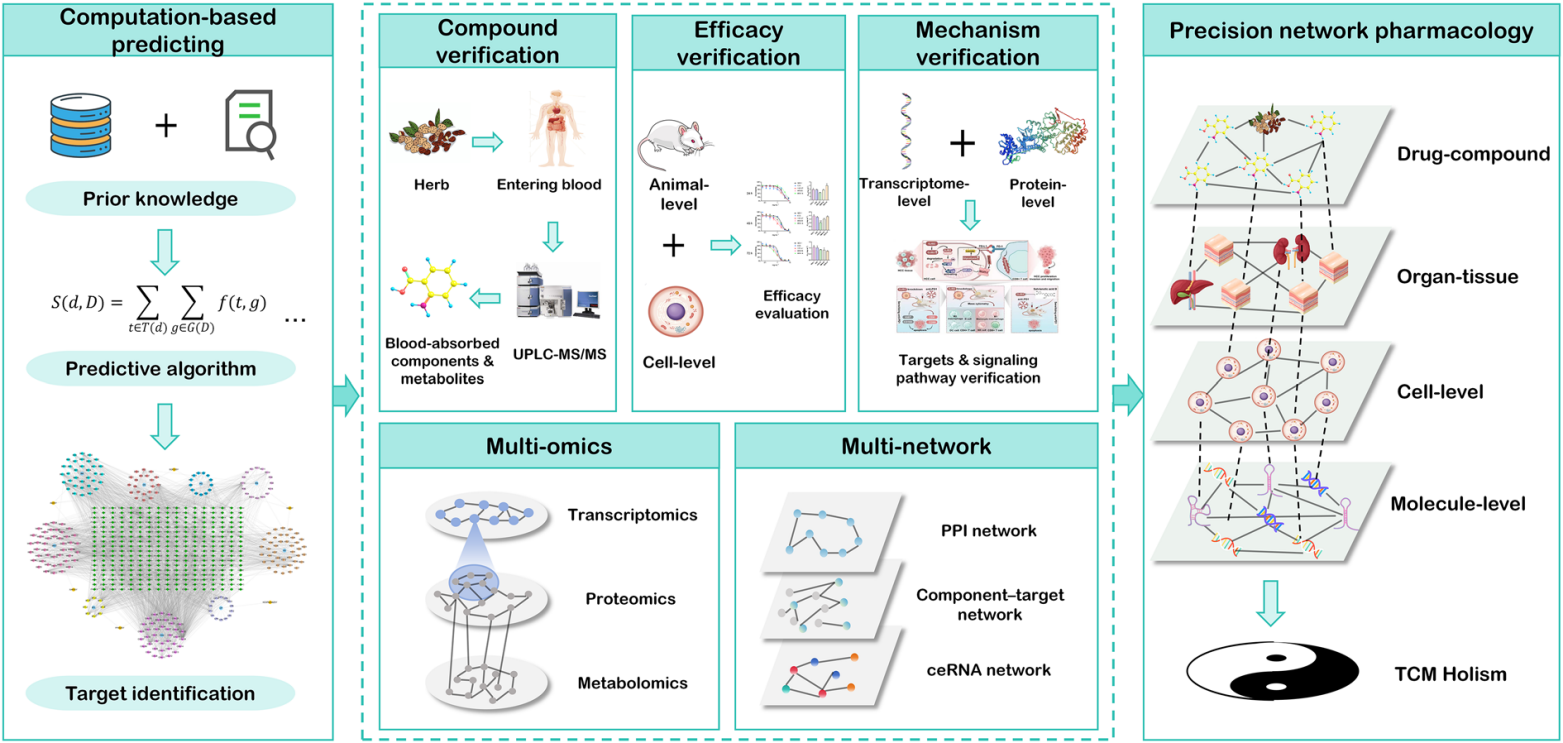
Schematic diagram of the paradigm of precision network pharmacology

Our team has synthesized existing methodologies in network pharmacology and proposed a “precision network pharmacology” research model [104] characterized by “triple verifications and dual integrations” (Fig. 3). The “triple verifications” encompass the verification of drug components, overall efficacy, and molecular mechanisms. Component verification involves employing suitable experimental techniques to identify and quantify the constituents of the drug that enter the bloodstream, along with their metabolic products; overall efficacy verification using animal and cellular experiments to confirm both in vivo and in vitro therapeutic effects; and molecular mechanism verification employs molecular biology experiments to verify the relevant targets and pathways identified through network pharmacology at the protein and RNA levels. The “dual integrations” refer to the integration of multi-omics and multi-network approaches. Multi-omics integration means that, alongside network pharmacology, methodologies such as proteomics, transcriptomics, and metabolomics should be employed to conduct a comprehensive analysis of the drug’s molecular mechanisms across different scales. Multi-network integration signifies the integration of classical network pharmacology constructs—such as PPI networks and component–target networks—with ceRNA networks, weighted gene co-expression networks, and other relevant frameworks. Chen et al. [105, 106], building on prior research into the chemical composition of Si-Miao-Yong-An Decoction (SMYAD), identified 130 compounds within the formula. They subsequently analyzed the blood-absorbed components involved in SMYAD’s intervention in myocardial hypertrophy, detecting 32 prototype compounds and 59 metabolites in the plasma and heart tissue of rats.

This research model provides a standardized, applicable workflow for network pharmacology analysis. Initially, LC–MS is employed to identify the chemical components of the herbs, yielding the prototype constituents. Subsequently, blood or tissue samples are collected from normal animals as well as those post-administration. By analyzing the component differences in plasma before and after administration, LC–MS identifies all absorbed constituents and their metabolites. Finally, a comparison with the previously identified herbal components reveals both the prototype absorbed components and their metabolic products. These absorbed constituents are then used to construct a component–target interaction network. Once the active constituents of the herbs are identified, their efficacy is confirmed through both in vivo and in vitro experiments. Using multi-omics approaches, the molecular mechanisms of the herbs are further explored through GO and KEGG enrichment analyses. By integrating target information obtained from multi-omics with network pharmacology’s PPI network and compound–target network, the molecular mechanisms underlying the herbal effects can be clarified. Finally, molecular biology techniques verify these mechanisms at both the protein and transcriptional levels. The precision network pharmacology model offers a more standardized framework for network pharmacology research.

In conclusion, future research on network pharmacology within TCM is expected to evolve further while preserving its unique characteristics. It is reasonable to anticipate that precision-driven network pharmacology will define the future trajectory of TCM network pharmacology, emerging as the predominant research paradigm in the field. As network pharmacology continues to intersect with disciplines such as life sciences, TCM, and artificial intelligence, it is poised for significant innovation in both its algorithms and experimental methodologies. These advancements will enable a more comprehensive and precise elucidation of the intricate chemical composition, mechanisms of action, and therapeutic targets of TCM.

## Data Availability

Not applicable.

## Abbreviations

AI: Artificial intelligence
BATMAN-TCM: A bioinformatics analysis tool for molecular mechanism of traditional Chinese medicine
CRP: Citri reticulatae pericarpium
CTD: Comparative toxicogenomics database
DEGs: Differentially expressed genes
DGIdb: Drug–gene interaction database
DL: Drug similarity
ETCM: The encyclopedia of traditional Chinese medicine
GO: Gene ontology
HERB: A high-throughput experiment-and reference-guided database of traditional Chinese medicine
HIT: Herbal ingredients’ targets database
HPLC–MS: High-performance liquid chromatography-mass spectrometry
HPRD: Human protein reference database
INR: Immunological non-responders
KEGG: Kyoto encyclopedia of genes and genomes
LC–MS: Liquid chromatography-mass spectrometry
LOS: Lanqin oral solution
MCP: Maca compounds prescription
MINT: Molecular interaction database
OB: Oral bioavailability
OMIM: Online Mendelian inheritance in man
PPI: Protein–protein interaction
PTMs: Post-translational modifications
SMYAD: Si-Miao-Yong-An decoction
STTTCH: Search tool for interactions of chemicals dataset
SymMap: Symptom mapping
TCM: Traditional Chinese medicine
TCM-ID: Traditional Chinese medicine information database
TCMID: Traditional Chinese medicine integrative database
TCMSP: Traditional Chinese medicine systems pharmacology database and analysis platform
TL: Thymus linearis benth
TwHF: Tripterygium Wilfordii Hook F
XCD: Xuanbai Chengqi decoction
XDT: Xijiao Dihuang Tang
ZGBSF: Zhuanggu Busui formula

## References

[1] Hopkins AL. Network pharmacology. Nat Biotechnol. 2007;25(10):1110–1. 17921993 10.1038/nbt1007-1110

[2] Hopkins AL. Network pharmacology: the next paradigm in drug discovery. Nat Chem Biol. 2008;4(11):682–90. 18936753 10.1038/nchembio.118

[3] Boezio B, Audouze K, Ducrot P, Taboureau O. Network-based approaches in pharmacology. Mol Inform. 2017;36(10):1700048–58. 10.1002/minf.201700048 28692140

[4] Greene JA, Loscalzo J. Putting the patient back together — social medicine, network medicine, and the limits of reductionism. N Engl J Med. 2017;377(25):2493–9. 29262277 10.1056/NEJMms1706744

[5] Nogales C, Mamdouh ZM, List M, Kiel C, Casas AI, Schmidt HHHW. Network pharmacology: curing causal mechanisms instead of treating symptoms. Trends Pharmacol Sci. 2022;43(2):136–50. 34895945 10.1016/j.tips.2021.11.004

[6] Assenov Y, Ramírez F, Schelhorn SE, Lengauer T, Albrecht M. Computing topological parameters of biological networks. Bioinformatics. 2007;24(2):282–4. 18006545 10.1093/bioinformatics/btm554

[7] Jiao X, Jin X, Ma Y, Yang Y, Li J, Liang L, et al. A comprehensive application: molecular docking and network pharmacology for the prediction of bioactive constituents and elucidation of mechanisms of action in component-based Chinese medicine. Comput Biol Chem. 2021;90: 107402. 33338839 10.1016/j.compbiolchem.2020.107402

[8] Yang M, Chen J, Xu LW, Ji G. Navigating traditional Chinese medicine network pharmacology and computational tools. Evid Based Complement Alternat Med. 2013;2013:1–23. 10.1155/2013/731969 PMC3747450 23983798

[9] Jiang M, Lu C, Zhang C, Yang J, Tan Y, Lu A, et al. Syndrome differentiation in modern research of traditional Chinese medicine. J Ethnopharmacol. 2012;140(3):634–42. 22322251 10.1016/j.jep.2012.01.033

[10] Li S, Zhang B. Traditional Chinese medicine network pharmacology: theory, methodology and application. Chin J Nat Med. 2013;11(2):110–20. 23787177 10.1016/S1875-5364(13)60037-0

[11] Li S. Correlation between TCM syndromes and molecular network regulation mechanism. The First Annual Conference of China Association for Science and Technology. Hangzhou; 1999 519

[12] Li S, Wang Y. A discussion and case study of complexities in traditional Chinese medicine. J System Simul. 2002;14(11):1429–1403.

[13] Li S. Network systems underlying traditional Chinese medicine syndrome and herb formula. Curr Bioinform. 2009;4(3):188–96.

[14] Shao L. Network target: a starting point for traditional Chinese medicine network pharmacology. China J Chin Materia Med. 2011;36(15):2017–20. 22066431

[15] Xu F, Cai W, Ma T, Zeng H, Kuang X, Chen W, et al. Traditional uses, phytochemistry, pharmacology, quality control, industrial application, pharmacokinetics and network pharmacology of Pogostemon cablin: A comprehensive review. Am J Chin Med. 2022;50(3):1–31. 35282804 10.1142/S0192415X22500288

[16] Wu J, Zhang F, Liu Z, Wu J, Shi Y. Integration strategy of network pharmacology in traditional Chinese medicine: a narrative review. J Tradit Chin Med. 2022;42(3):479–86. 35610020 10.19852/j.cnki.jtcm.20220408.003 PMC9924699

[17] Zhai Y, Zhang F, Zhou J, Qiao C, Jin Z, Zhang J, et al. Mechanism of norcantharidin intervention in gastric cancer: analysis based on antitumor proprietary Chinese medicine database, network pharmacology, and transcriptomics. Chin Med. 2024;19(1):129. 39289763 10.1186/s13020-024-01000-1 PMC11406961

[18] Dai Y, Chen X, Yang H, Yang J, Hu Q, Xiao X, et al. Evidence construction of Huangkui capsule against chronic glomerulonephritis: a systematic review and network pharmacology. Phytomedicine. 2022;102: 154189. 35617887 10.1016/j.phymed.2022.154189

[19] Li N, Gu X, Liu F, Zhang Y, Sun Y, Gao S, et al. Network pharmacology-based analysis of potential mechanisms of myocardial ischemia-reperfusion injury by total salvianolic acid injection. Front Pharmacol. 2023;14:1202748. 10.3389/fphar.2023.1202718 PMC10482107 37680709

[20] Zhang R, Zhu X, Bai H, Ning K. Network pharmacology databases for traditional Chinese medicine: Review and assessment. Front Pharmacol. 2019;10:123. 30846939 10.3389/fphar.2019.00123 PMC6393382

[21] Ru J, Li P, Wang J, Zhou W, Li B, Huang C, et al. TCMSP: a database of systems pharmacology for drug discovery from herbal medicines. J Cheminform. 2014;6(1):13. 24735618 10.1186/1758-2946-6-13 PMC4001360

[22] Xu HY, Zhang YQ, Liu ZM, Chen T, Lv CY, Tang SH, et al. ETCM: an encyclopaedia of traditional Chinese medicine. Nucleic Acids Res. 2019;47(D1):D976–82. 30365030 10.1093/nar/gky987 PMC6323948

[23] Zhang Y, Li X, Shi Y, Chen T, Xu Z, Wang P, et al. ETCM v20: An update with comprehensive resource and rich annotations for traditional Chinese medicine. Acta Pharm Sin B. 2023;13(6):2559–71. 37425046 10.1016/j.apsb.2023.03.012 PMC10326295

[24] Wu Y, Zhang F, Yang K, Fang S, Bu D, Li H, et al. SymMap: an integrative database of traditional Chinese medicine enhanced by symptom mapping. Nucleic Acids Res. 2019;47(D1):D1110–7. 30380087 10.1093/nar/gky1021 PMC6323958

[25] Kong X, Liu C, Zhang Z, Cheng M, Mei Z, Li X, et al. BATMAN-TCM 2.0: an enhanced integrative database for known and predicted interactions between traditional Chinese medicine ingredients and target proteins. Nucleic Acids Res. 2023;52(D1):D1110-20. 10.1093/nar/gkad926 PMC10767940 37904598

[26] Xue R, Fang Z, Zhang M, Yi Z, Wen C, Shi T. TCMID: traditional Chinese medicine integrative database for herb molecular mechanism analysis. Nucleic Acids Res. 2013;41(D1):D1089–95. 23203875 10.1093/nar/gks1100 PMC3531123

[27] Fang S, Dong L, Liu L, Guo JC, Zhao L, Zhang JY, et al. HERB: a high-throughput experiment- and reference-guided database of traditional Chinese medicine. Nucleic Acids Res. 2021;49(D1):D1197–206. 33264402 10.1093/nar/gkaa1063 PMC7779036

[28] Chen CYC. TCM Database@Taiwan: The world’s largest traditional Chinese medicine database for drug screening in silico. PLoS ONE. 2011;6(1): e15939. 21253603 10.1371/journal.pone.0015939 PMC3017089

[29] Wang J, Zhou H, Han L, Chen X, Chen Y, Cao Z. Traditional Chinese medicine information database. Clin Pharmacol Ther. 2005;78(1):92–3. 16003299 10.1016/j.clpt.2005.03.010

[30] Yan D, Zheng G, Wang C, Chen Z, Mao T, Gao J, et al. HIT 2.0: an enhanced platform for Herbal Ingredients’ Targets. Nucleic Acids Res. 2022;50(D1):D1238-43. 34986599 10.1093/nar/gkab1011 PMC8728248

[31] Kim S, Chen J, Cheng T, Gindulyte A, He J, He S, et al. PubChem 2023 update. Nucleic Acids Res. 2023;51(D1):D1373–80. 36305812 10.1093/nar/gkac956 PMC9825602

[32] Daina A, Michielin O, Zoete V. SwissADME: a free web tool to evaluate pharmacokinetics, drug-likeness and medicinal chemistry friendliness of small molecules. Sci Rep. 2017;7(1):1–13. 28256516 10.1038/srep42717 PMC5335600

[33] Zdrazil B, Félix E, Hunter F, Manners E, Blackshaw J, Corbett S, et al. The ChEMBL Database in 2023: a drug discovery platform spanning multiple bioactivity data types and time periods. Nucleic Acids Res. 2024;52(D1):D1180–92. 37933841 10.1093/nar/gkad1004 PMC10767899

[34] Cannon M, Stevenson JG, Stahl K, Basu RK, Coffman AC, Kiwala S, et al. DGIdb 50: rebuilding the drug–gene interaction database for precision medicine and drug discovery platforms. Nucleic Acids Res. 2024;52(D1):D1227-35. 37953380 10.1093/nar/gkad1040 PMC10767982

[35] Davis AP, Wiegers TC, Johnson RJ, Sciaky D, Wiegers J, Mattingly C. Comparative toxicogenomics database (CTD): update 2023. Nucleic Acids Res. 2023;51(D1):D1257–62. 36169237 10.1093/nar/gkac833 PMC9825590

[36] Szklarczyk D, Franceschini A, Wyder S, Forslund K, Heller D, Huerta-Cepas J, et al. STRING v10: protein-protein interaction networks, integrated over the tree of life. Nucleic acids res. 2015;43(D1):D447–52. 25352553 10.1093/nar/gku1003 PMC4383874

[37] Avram S, Wilson TB, Curpăn R, Halip L, Borota A, Bora A, et al. DrugCentral 2023 extends human clinical data and integrates veterinary drugs. Nucleic Acids Res. 2023;51(D1):D1276–87. 36484092 10.1093/nar/gkac1085 PMC9825566

[38] Knox C, Wilson M, Klinger CM, Franklin M, Oler E, Wilson A, et al. DrugBank 60: the DrugBank knowledgebase for 2024. Nucleic Acids Res. 2024;52(D1):D1265-75. 37953279 10.1093/nar/gkad976 PMC10767804

[39] Piñero J, Ramírez-Anguita JM, Saüch-Pitarch J, Ronzano F, Centeno E, Sanz F, et al. The DisGeNET knowledge platform for disease genomics: 2019 update. Nucleic Acids Res. 2020;48(D1):D845–55. 31680165 10.1093/nar/gkz1021 PMC7145631

[40] Stelzer G, Rosen N, Plaschkes I, Zimmerman S, Twik M, Fishilevich S, et al. The GeneCards Suite From gene data mining to disease genome sequence analyses. Curr Protoc Bioinformatics. 2016. 10.1002/cpbi.5. 27322403 10.1002/cpbi.5

[41] Amberger JS, Bocchini CA, Schiettecatte F, Scott AF, Hamosh A. OMIMorg: online mendelian inheritance in man (OMIM®), an online catalog of human genes and genetic disorders. Nucleic Acids Res. 2014;43(D1):D789-98. 25428349 10.1093/nar/gku1205 PMC4383985

[42] Peri S, Navarro JD, Kristiansen TZ, Amanchy R, Surendranath V, Muthusamy B, et al. Human protein reference database as a discovery resource for proteomics. Nucleic Acids Res. 2004;32(D1):D497-501. 14681466 10.1093/nar/gkh070 PMC308804

[43] Szklarczyk D, Kirsch R, Koutrouli M, Nastou K, Mehryary F, Hachilif R, et al. The STRING database in 2023: protein–protein association networks and functional enrichment analyses for any sequenced genome of interest. Nucleic Acids Res. 2023;51(D1):D638–46. 36370105 10.1093/nar/gkac1000 PMC9825434

[44] Burley SK, Bhikadiya C, Bi C, Bittrich S, Chao H, Chen L, et al. RCSB Protein Data Bank (RCSBorg) delivery of experimentally-determined PDB structures alongside one million computed structure models of proteins from artificial intelligence/machine learning. Nucleic Acids Res. 2022;51(D1):D488-508. 10.1093/nar/gkac1077 PMC9825554 36420884

[45] Sherman BT, Hao M, Qiu J, Jiao X, Baseler MW, Lane HC, et al. DAVID: a web server for functional enrichment analysis and functional annotation of gene lists (2021 update). Nucleic Acids Res. 2022;50(W1):W216–21. 35325185 10.1093/nar/gkac194 PMC9252805

[46] Zhou Y, Zhou B, Pache L, Chang M, Khodabakhshi AH, Tanaseichuk O, et al. Metascape provides a biologist-oriented resource for the analysis of systems-level datasets. Nat Commun. 2019;10(1):1523. 30944313 10.1038/s41467-019-09234-6 PMC6447622

[47] Licata L, Briganti L, Peluso D, Perfetto L, Iannuccelli M, Galeota E, et al. MINT, the molecular interaction database: 2012 update. Nucleic Acids Res. 2012;40(D1):D857–61. 22096227 10.1093/nar/gkr930 PMC3244991

[48] Kanehisa M, Furumichi M, Sato Y, Kawashima M, Ishiguro-Watanabe M. KEGG for taxonomy-based analysis of pathways and genomes. Nucleic Acids Res. 2023;51(D1):D587–92. 36300620 10.1093/nar/gkac963 PMC9825424

[49] Zhao L, Zhang H, Li N, Chen J, Xu H, Wang Y, et al. Network pharmacology, a promising approach to reveal the pharmacology mechanism of Chinese medicine formula. J Ethnopharmacol. 2023;309: 116306. 36858276 10.1016/j.jep.2023.116306

[50] Maayan A, Jenkins SL, Goldfarb J, Iyengar R. Network analysis of FDA approved drugs and their targets. Mt Sinai J Med. 2007;74(1):27–32. 17516560 10.1002/msj.20002 PMC2561141

[51] Wang YL, Liang YZ, Chen BM, He YK, Li BY, Hu QN. LC-DAD-APCI-MS-based screening and analysis of the absorption and metabolite components in plasma from a rabbit administered an oral solution of danggui. Anal Bioanal Chem. 2005;383(2):247–54. 16132135 10.1007/s00216-005-0008-7

[52] Hou F, Yu Z, Cheng Y, Liu Y, Liang S, Zhang F. Deciphering the pharmacological mechanisms of Scutellaria baicalensis Georgi on oral leukoplakia by combining network pharmacology, molecular docking and experimental evaluations. Phytomedicine. 2022;103: 154195. 35667260 10.1016/j.phymed.2022.154195

[53] Lou N, Zhai M, Su Z, Chu F, Li Y, Chen Y, et al. Pharmacodynamics and pharmacological mechanism of Moluodan concentrated pill in the treatment of atrophic gastritis: a network pharmacological study and in vivo experiments. J Ethnopharmacol. 2024;318: 116937. 37480968 10.1016/j.jep.2023.116937

[54] Thakur P, Kumar R, Choudhary N, Sharma R, Chaudhary A. Network pharmacology on mechanistic role of Thymus linearis Benth against gastrointestinal and neurological diseases. Phytomedicine. 2023;121: 155098. 37757710 10.1016/j.phymed.2023.155098

[55] Cao X, Shi K, Xu Y, Zhang P, Zhang H, Pan S. Integrated metabolomics and network pharmacology to reveal antioxidant mechanisms and potential pharmacological ingredients of citrus herbs. Food Res Int. 2023;174: 113514. 37986422 10.1016/j.foodres.2023.113514

[56] Yan Y, Li J, Zhang Y, Wang H, Qin X, Zhai K, et al. Screening the effective components of Suanzaoren decoction on the treatment of chronic restraint stress induced anxiety-like mice by integrated chinmedomics and network pharmacology. Phytomedicine. 2023;115: 154853. 37156059 10.1016/j.phymed.2023.154853

[57] Chen Y, Dai Y, Xia J, Liu J, Zhou G, Chen C, et al. Serum pharmacochemistry combining network pharmacology to discover the active constituents and effect of Xijiao Dihuang Tang prescription for treatment of blood-heat and blood-stasis syndrome-related disease. Oxid Med Cell Longev. 2022;2022(1):6934812. 35178159 10.1155/2022/6934812 PMC8845118

[58] Bai L, Wu C, Lei S, Zou M, Wang S, Zhang Z, et al. Potential anti-gout properties of Wuwei Shexiang pills based on network pharmacology and pharmacological verification. J Ethnopharmacol. 2023;305: 116147. 36608779 10.1016/j.jep.2023.116147

[59] Zhu H, Wang R, Hua H, Cheng Y, Guo Y, Qian H, et al. Network pharmacology exploration reveals gut microbiota modulation as a common therapeutic mechanism for anti-fatigue effect treated with Maca compounds prescription. Nutrients. 2022;14(8):1533. 35458095 10.3390/nu14081533 PMC9026883

[60] Zhao M, Fu L, Xu P, Wang T, Li P. Network pharmacology and experimental validation to explore the effect and mechanism of Kanglaite Injection against triple-negative breast cancer. Drug Des Devel Ther. 2023;17:901–17. 36998242 10.2147/DDDT.S397969 PMC10043292

[61] Shi P, Chen J, Ge W, Liu Z, Han N, Yin J. Antichilblain components in eggplant based on network pharmacology and biological evaluation. J Agric Food Chem. 2023;71(30):11442–53. 37467304 10.1021/acs.jafc.3c01108

[62] Zeng L, Yang K. Exploring the pharmacological mechanism of Yanghe Decoction on HER2-positive breast cancer by a network pharmacology approach. J Ethnopharmacol. 2017;199:68–85. 28130113 10.1016/j.jep.2017.01.045

[63] Li P, Su W. Recent progress in applying network pharmacology to research of Chinese materia medica. Chin Tradit Herbal Drugs. 2016;47(16):2938–42.

[64] Shang L, Wang Y, Li J, Zhou F, Xiao K, Liu Y, et al. Mechanism of Sijunzi Decoction in the treatment of colorectal cancer based on network pharmacology and experimental validation. J Ethnopharmacol. 2022;302: 115876. 36343798 10.1016/j.jep.2022.115876

[65] Zhou W, Zhang H, Wang X, Kang J, Guo W, Zhou L, et al. Network pharmacology to unveil the mechanism of Moluodan in the treatment of chronic atrophic gastritis. Phytomedicine. 2022;95: 153837. 34883416 10.1016/j.phymed.2021.153837

[66] Chen Y, Li K, Zhao H, Hao Z, Yang Y, Gao M, et al. Integrated lipidomics and network pharmacology analysis to reveal the mechanisms of berberine in the treatment of hyperlipidemia. J Transl Med. 2022;20(1):412. 36076294 10.1186/s12967-022-03623-0 PMC9461205

[67] Jiao W, Mi S, Sang Y, Jin Q, Chitrakar Bimal, Wang X, et al. Integrated network pharmacology and cellular assay for the investigation of an anti-obesity effect of 6-shogaol. Food Chem. 2021;374:131755. 34883426 10.1016/j.foodchem.2021.131755

[68] Zhang H, Zhou C, Zhang Z, Yao S, Bian Y, Fu F, et al. Integration of network pharmacology and experimental validation to explore the pharmacological mechanisms of Zhuanggu Busui formula against osteoporosis. Front Endocrinol. 2022;12: 841668. 10.3389/fendo.2021.841668 PMC8831245 35154014

[69] Zhu H, Wang S, Shan C, Li X, Tan B, Chen Q, et al. Mechanism of protective effect of xuan-bai-cheng-qi decoction on LPS-induced acute lung injury based on an integrated network pharmacology and RNA-sequencing approach. Respir Res. 2021;22(1):188. 34183011 10.1186/s12931-021-01781-1 PMC8237774

[70] Wang T, Jiang X, Ruan Y, Zhuang J, Yin Y. Based on network pharmacology and in vitro experiments to prove the effective inhibition of myocardial fibrosis by Buyang Huanwu decoction. Bioengineered. 2022;13(5):13767–83. 35726821 10.1080/21655979.2022.2084253 PMC9275964

[71] Xiong M, Chen X, Wang H, Tang X, Wang Q, Li X, et al. Combining transcriptomics and network pharmacology to reveal the mechanism of Zuojin capsule improving spasmolytic polypeptide-expressing metaplasia. J Ethnopharmacol. 2024;318: 117075. 37625606 10.1016/j.jep.2023.117075

[72] Li J, Wang Y, Ullah A, Zhang R, Sun Y, Li J, et al. Network pharmacology and molecular modeling techniques in unraveling the underlying mechanism of Citri Reticulatae Pericarpium aganist type 2 diabetic osteoporosis. Nutrients. 2024;16(2):220. 38257113 10.3390/nu16020220 PMC10819846

[73] Kong J, Sun S, Min F, Hu X, Zhang Y, Cheng Y, et al. Integrating network pharmacology and transcriptomic strategies to explore the pharmacological mechanism of hydroxysafflor yellow a in delaying liver aging. Int J Mol Sci. 2022;23(22):14281. 36430769 10.3390/ijms232214281 PMC9697017

[74] Huang Z, Wu C, Zhou W, Lu S, Tan Y, Wu Z, et al. Compound Kushen Injection inhibits epithelial-mesenchymal transition of gastric carcinoma by regulating VCAM1 induced by the TNF signaling pathway. Phytomedicine. 2023;118: 154984. 37487253 10.1016/j.phymed.2023.154984

[75] Zhang C, Xu Y, Tan H, Li S, Wang N, Zhang Y, et al. Neuroprotective effect of He-Ying-Qing-Re formula on retinal ganglion cell in diabetic retinopathy. J Ethnopharmacol. 2018;214:179–89. 29253613 10.1016/j.jep.2017.12.018

[76] Fan X, Zhao X, Jin Y, Shen X, Liu C. Network toxicology and its application to traditional Chinese medicine. China J Chin Materia Med. 2021;36(21):2920–2. 22308674

[77] Yin ZQ, Luo D, Ma L, Xu J, Xia J. Pustular drug eruption due to Panax notoginseng saponins. Drug Des Devel Ther. 2014;8:957–61. 25114505 10.2147/DDDT.S67015 PMC4109629

[78] Shen B, Guo K, Guo Y, Kong J, Liu C. Research status and development thinking of network toxicology in safety evaluation of traditional Chinese medicine. Drug Evaluation Research. 2024;47(1):179–90.

[79] Liao Y, Zhao K, Guo H. Application and challenges of network pharmacology research in traditional Chinese medicine. Chinese Tradit Herbal Drugs. 2024;55(12):4204–13.

[80] Zhang C, Wang N, Xu Y, Tan HY, Li S, Feng Y. Molecular mechanisms involved in oxidative stress-associated liver injury induced by Chinese herbal medicine: an experimental evidence-based literature review and network pharmacology study. Int J Mol Sci. 2018;19(9):2745. 30217028 10.3390/ijms19092745 PMC6165031

[81] Jiang HY, Gao HY, Li J, Zhou TY, Wang ST, Yang JB, et al. Integrated spatially resolved metabolomics and network toxicology to investigate the hepatotoxicity mechanisms of component D of Polygonum multiflorum Thunb. J Ethnopharmacol. 2022;298: 115630. 35987407 10.1016/j.jep.2022.115630

[82] Cao D, Zhao C, Li Z, Fan Q, Chen M, Jiang Y, et al. Combined metabolomics and network toxicology to explore the molecular mechanism of Phytolacca acinose Roxb-induced hepatotoxicity in Zebrafish Larvae in Vivo. Evid Based Complement Alternat Med. 2021;2021:3303014. 34876912 10.1155/2021/3303014 PMC8645354

[83] He T, Liu C, Li M, Wang M, Liu N, Zhang D, et al. Integrating non-targeted metabolomics and toxicology networks to study the mechanism of Esculentoside a-induced hepatotoxicity in rats. J Biochem Mol Toxicol. 2021;35(6):1–15. 33788351 10.1002/jbt.22761

[84] Chen Q, Zhang K, Jiao M, Jiao J, Chen D, Yin Y, et al. Study on the mechanism of Mesaconitine-induced hepatotoxicity in rats based on metabonomics and toxicology network. Toxins. 2022;14(7):486. 35878224 10.3390/toxins14070486 PMC9322933

[85] Casas AI, Hassan AA, Larsen SJ, Gomez-Rangel V, Elbatreek M, Kleikers PWM, et al. From single drug targets to synergistic network pharmacology in ischemic stroke. Proc Natl Acad Sci. 2019;116(14):7129–36. 30894481 10.1073/pnas.1820799116 PMC6452748

[86] Sun D, Gao W, Hu H, Zhou S. Why 90% of clinical drug development fails and how to improve it? Acta Pharm Sin B. 2022;12(7):3049–62. 35865092 10.1016/j.apsb.2022.02.002 PMC9293739

[87] Makhoba XH, Viegas C Jr, Mosa RA, Viegas FPD, Pooe OJ. Potential impact of the multi-target drug approach in the treatment of some complex diseases. Drug Des Devel Ther. 2020;14:3235–49. 32884235 10.2147/DDDT.S257494 PMC7440888

[88] Löscher W. Single-target versus multi-target drugs versus combinations of drugs with multiple targets: Preclinical and clinical evidence for the treatment or prevention of epilepsy. Front Pharmacol. 2021;12: 730257. 34776956 10.3389/fphar.2021.730257 PMC8580162

[89] Löscher W, Klein P. New approaches for developing multi-targeted drug combinations for disease modification of complex brain disorders. Does epilepsy prevention become a realistic goal? Pharmacol Ther. 2021;229: 107934. 34216705 10.1016/j.pharmthera.2021.107934

[90] Zhang H, Zhang Y, Li Y, Wang Y, Yan S, Xu S, et al. Bioinformatics and network pharmacology identify the therapeutic role and potential mechanism of melatonin in AD and rosacea. Front Immunol. 2021;12: 756550. 34899707 10.3389/fimmu.2021.756550 PMC8657413

[91] Zhang DY, Peng RQ, Wang X, Zuo HL, Lyu LY, Yang FQ, et al. A network pharmacology-based study on the quality control markers of antithrombotic herbs: using Salvia miltiorrhiza - Ligusticum chuanxiong as an example. J Ethnopharmacol. 2022;292: 115197. 35331879 10.1016/j.jep.2022.115197

[92] Ma H, Fu W, Yu H, Xu Y, Xiao L, Zhang Y, et al. Exploration of the anti-inflammatory mechanism of Lanqin oral solution based on the network pharmacology analysis optimized by Q-markers selection. Comput Biol Med. 2023;154: 106607. 36731363 10.1016/j.compbiomed.2023.106607

[93] Li S, Xiao W, Niu M, Sun D, Zhang P, Zhang B, et al. Expert consensus on application of network pharmacology in research and development of new traditional Chinese medicine drugs. China J Chinese Materia Medica. 2024. 10.19540/j.cnki.cjcmm.20240818.701. 10.19540/j.cnki.cjcmm.20240818.701 39701688

[94] Li L, Yang L, Yang L, He C, He Y, Chen L, et al. Network pharmacology: a bright guiding light on the way to explore the personalized precise medication of traditional Chinese medicine. Chin Med. 2023;18(1):146. 37941061 10.1186/s13020-023-00853-2 PMC10631104

[95] Wang Y, Song F, Xu Q, Liu Z, Liu S. Characterization of aconitine-type alkaloids in the flowers ofAconitum kusnezoffii by electrospray ionization tandem mass spectrometry. J Mass Spectrom. 2003;38(9):962–70. 14505324 10.1002/jms.510

[96] Li S. Network pharmacology evaluation method guidance - Draft. World J Tradit Chinese Med. 2021;7(1):146–54.

[97] Noor F, Asif M, Ashfaq UA, Qasim M, Tahir QM. Machine learning for synergistic network pharmacology: a comprehensive overview. Brief Bioinform. 2023. 10.1093/bib/bbad120. 37031957 10.1093/bib/bbad120

[98] Zhang P, Zhang D, Zhou W, Wang L, Wang B, Zhang T, et al. Network pharmacology: towards the artificial intelligence-based precision traditional Chinese medicine. Brief Bioinform. 2023. 10.1093/bib/bbad518. 38197310 10.1093/bib/bbad518 PMC10777171

[99] Keiser MJ, Setola V, Irwin JJ, Laggner C, Abbas A, Hufeisen SJ, et al. Predicting new molecular targets for known drugs. Nature. 2009;462(7270):175–81. 19881490 10.1038/nature08506 PMC2784146

[100] Zhao S, Li S. Network-based relating pharmacological and genomic spaces for drug target identification. PLoS ONE. 2010. 10.1371/journal.pone.0011764. 20668676 10.1371/journal.pone.0011764 PMC2909904

[101] Lv S, Wang Q, Zhang X, Ning F, Liu W, Cui M, et al. Mechanisms of multi-omics and network pharmacology to explain traditional chinese medicine for vascular cognitive impairment: a narrative review. Phytomedicine. 2024;123: 155231. 38007992 10.1016/j.phymed.2023.155231

[102] Ye J, Huang F, Zeng H, Xu X, Wu G, Tian S, et al. Multi-omics and network pharmacology study reveals the effects of Dengzhan Shengmai capsule against neuroinflammatory injury and thrombosis induced by ischemic stroke. J Ethnopharmacol. 2023;305: 116092. 36587875 10.1016/j.jep.2022.116092

[103] Liu X, Lin L, Lv T, Lu L, Li X, Han Y, et al. Combined multi-omics and network pharmacology approach reveals the role of Tripterygium Wilfordii Hook F in treating HIV immunological non-responders. Phytomedicine. 2022;101: 154103. 35468451 10.1016/j.phymed.2022.154103

[104] Wu J. A precision network pharmacology investigation into the anti-tumor mechanisms of Compound Kushen Injection. Beijing: China Press of Chinese Medicine; 2024.

[105] Ren Y, Chen X, Li P, Zhang H, Su C, Zeng Z, et al. Si-Miao-Yong-An decoction ameliorates cardiac function through restoring the equilibrium of SOD and NOX2 in heart failure mice. Pharmacol Res. 2019;146: 104318. 31228552 10.1016/j.phrs.2019.104318

[106] Chen XY, Chen XH, Li L, Su CP, Zhang YL, Jiang YY, et al. Deciphering the effective combinatorial components from Si-Miao-Yong-An decoction regarding the intervention on myocardial hypertrophy. J Ethnopharmacol. 2021;271: 113833. 33465437 10.1016/j.jep.2021.113833

